# Molecular characterization of a novel *Trichinella spiralis* aspartyl protease and its role in the larval invasion of host intestinal epithelial cells

**DOI:** 10.1051/parasite/2026050

**Published:** 2026-09-14

**Authors:** Yao Zhang, Ru Zhang, Xin Zhuo Zhang, Shao Rong Long, Xi Zhang, Ruo Dan Liu, Zhong Quan Wang, Jing Cui

**Affiliations:** Department of Parasitology, School of Basic Medical Sciences, Zhengzhou University Zhengzhou 450001 PR China

**Keywords:** *Trichinella spiralis*, Aspartyl protease, Larval invasion, Caco-2 cells

## Abstract

The aim of the present study was to characterize the molecular properties of a novel *Trichinella spiralis* aspartic protease (TsEasp) and to investigate its role in the larval invasion of host intestinal epithelial cells. The full-length TsEasp cDNA was cloned and expressed, and recombinant TsEasp protein (rTsEasp) was purified. Immunization of mice with rTsEasp elicited a robust specific IgG response. qPCR analyses revealed that TsEasp was transcribed at all *T. spiralis* developmental stages, with markedly higher transcription levels in intestinal infective larvae and adult worms. Indirect immunofluorescence testing showed that TsEasp was expressed throughout the *T. spiralis* life cycle, and mainly localized at the cuticle, and embryonic periphery in adult females. Far-western blot and indirect immunofluorescence confirmed that rTsEasp binds specifically to Caco-2 cells, predominantly on the cell membrane. The *in vitro* invasion assay showed that rTsEasp significantly accelerated the larval invasion of Caco-2 monolayers in a dose-dependent manner of rTsEasp protein, whereas anti-rTsEasp antibodies and RNAi markedly suppressed this invasion. Collectively, our results indicate that TsEasp is a key invasive virulence factor in *T. spiralis* infection.

## Abstract (fr)

L'objectif de cette étude était de caractériser les propriétés moléculaires d'une nouvelle aspartyl-protéase de *Trichinella spiralis* (TsEasp) et d'étudier son rôle dans l'invasion par les larves des cellules épithéliales intestinales de l'hôte. L'ADNc complet de TsEasp a été cloné et exprimé, et la protéine recombinante TsEasp (rTsEasp) a été purifiée. L'immunisation de souris avec la rTsEasp a induit une réponse robuste en IgG spécifiques. Les analyses par qPCR ont révélé que TsEasp était transcrite à tous les stades de développement de *T. spiralis*, avec des niveaux de transcription nettement plus élevés chez les larves infectieuses intestinales et les vers adultes. L'immunofluorescence indirecte a montré que TsEasp était exprimée tout au long du cycle biologique de *T. spiralis* et localisée principalement au niveau de la cuticule ainsi qu'à la périphérie des embryons chez les femelles adultes. Des analyses de type Far-Western blot et d’immunofluorescence indirecte ont confirmé que la rTsEasp se lie spécifiquement aux cellules Caco-2, principalement au niveau de la membrane cellulaire. Le test d'invasion *in vitro* a montré que la rTsEasp accélérait de manière significative l'invasion des monocouches de cellules Caco-2 par les larves, selon une relation dose-dépendante, tandis que les anticorps dirigés contre la rTsEasp et l'ARNi inhibaient fortement cette invasion. Dans l'ensemble, nos résultats indiquent que TsEasp constitue un facteur de virulence clé impliqué dans l'invasion lors de l'infection par *T. spiralis*.

## Introduction

Trichinellosis is a widespread zoonotic disease induced by nematodes belonging to the genus *Trichinella* spp. Transmission of *Trichinella* spp. infection to humans occurs primarily via consumption of raw or insufficiently cooked meat contaminated with infective larvae, with pork and wildlife meats being the major infection sources. *Trichinella* exhibits worldwide geographical distribution and can infect a broad spectrum of hosts through both domestic and sylvatic transmission cycles. The transmission patterns and epidemiologic features of trichinellosis are largely determined by the feeding behaviors of diverse hosts and the impacts of anthropogenic activities [11]. Despite being neglected for a long time, trichinellosis has impacted human health for centuries and remains a substantial global public health concern, especially in areas with inadequate food safety supervision and control systems [29]. In the European Union, 76 confirmed human cases were reported in 11 member states in 2023 [10]. Between 2009 and 2020, 479 cases of trichinellosis (including 2 deaths) were documented in China. Most cases occurred in ethnic minority areas with a tradition of consuming raw meat, and pork constituted the main source of infection [64].

Unlike most nematodes, the life cycle of *Trichinella spiralis* does not require development in the external environment, and all developmental stages are completed within a single host. After the host ingests raw or undercooked meat containing infective muscle larvae (ML), the ML are liberated from their capsules in the small intestine under the combined action of gastric and intestinal fluids, invade intestinal mucosal epithelial cells, and develop into adult worms (AWs) following four molts. After mating, the female AWs produce newborn larvae (NBL), which disseminate via the blood circulation to the skeletal muscles throughout the body. The larvae finally develop into infective ML within nurse cells of muscle fibers, awaiting ingestion by a subsequent host to complete the life cycle [8]. *Trichinella spiralis* infection poses a severe threat to public health and pork food safety [37, 47]. Nevertheless, no preventive commercial anti-*Trichinella* vaccines are available [44]. Therefore, the development of an anti-*Trichinella* vaccine is needed for the control and elimination of *Trichinella* infection in food animals [53].

Aspartic proteases are a class of highly conserved hydrolytic enzymes that exert dual core functions in pathogen infection and host immune regulation [5]. The aspartic protease family includes pepsin, renin, cathepsins D and E, and chymosins [46]. They are characterized by a typical Asp-Thr(Ser)-Gly sequence, and their hydrolysis activity was correlated with the Asp residue in the clefts of their active sites [45]. Aspartic protease proteolysis function is optimal in acidic conditions [2]. Many aspartic proteases have important functions in degrading host hemoglobin and other proteins, especially in blood parasites. In *Plasmodium falciparum*, these proteases are crucial for the degradation of hemoglobin and acquisition of amino acids during the erythrocytic stage, and can be specifically inhibited by pepstatin [4, 6, 16, 19]. In the nematode *Steinernema carpocapsae*, secreted aspartic proteases are highly expressed in the early stage of parasitism and facilitate parasite invasion and colonization by degrading host tissue proteins [2]. From the host side, cathepsin E, a member of the pepsin superfamily, participates in anti-infection and anti-tumor immunity by maintaining lysosomal pH and regulating antigen presentation, whereas cathepsin D is mainly involved in extracellular matrix degradation, tumor proliferation and invasion, as well as epidermal keratin metabolism [33]. Collectively, aspartic proteases not only supply pathogens with nitrogen sources and amino acids and mediate tissue invasion, but also trigger inflammatory responses by activating the host NLRP3 inflammasome pathway. As such, they serve as important virulence factors and key targets for development of anti-parasite drugs [5].

Aspartic protease is also a key invasion factor of *T. spiralis*. TsASP2, a major excretory–secretory effector, mediates larval invasion by binding intestinal epithelial cells and degrading tight junction proteins, and serves as a promising vaccine candidate against trichinellosis [25, 56, 57]. However, the function of its homolog TsASP1 remains unclear. Our previous study showed that TsASP1 is highly expressed in intestinal infective larvae (IIL) and contributes to parasite development and invasion. Silencing TsASP1 attenuated *T. spiralis* pathogenicity, while vaccination with rTsASP1 in mice induced partial immune protection, suggesting that TsASP1 was a valuable vaccine candidate [59].

In our previous studies, a novel *T. spiralis* aspartyl protease, TsEasp (GenBank: XM_003373265.1), belonging to the eukaryotic aspartyl protease superfamily, was identified in the IIL stage [20, 30, 35]. However, the biological characteristics of TsEasp and its functions in the *T. spiralis* life-cycle are unclear. The purpose of this study was to clone and express TsEasp, to characterize its biological features, and to ascertain its function in the larval invasion of the enteral epithelium.

## Materials and methods

### Ethics statement

This study was performed in accordance with the National Guidelines for Experimental Animal Welfare (Minister of Science and Technology, People’s Republic of China, 2006). All animal experiments in our research were approved by the Life Science Ethics Committee of Zhengzhou University (No. ZZUIRB GZR 2023-1397).

### *Trichinella* isolate, cell line and mice

The *Trichinella* species employed in this study was *T. spiralis* isolate ISS534, originally recovered from a naturally infected domestic pig in Nanyang, Henan Province, PR China [50]. Human colon epithelial cell line Caco-2 was obtained from the Cell Resource Center of the Shanghai Institute for Biological Sciences of the Chinese Academy of Sciences. Lastly, 6–8-week-old female BALB/c mice were obtained from the Experimental Animal Center of Zhengzhou University.

### Collection of *T. spiralis* at different developmental stages and protein preparation

Mice were infected with *T. spiralis* for 35 days, followed by digestion of murine muscles with artificial gastric juice (1% pepsin, 0.75% HCl, and 0.9% NaCl) at 37 °C for 3 h [17]. The IIL and AWs were isolated from the small intestines of infected mice at different time points after infection [36, 39]. The 6-day-old AWs (6 d AW) were washed with sterile PBS and cultured in serum-free RPMI-1640 (Servicebio, Wuhan, PR China) 37 °C for 24 h, after which the NBL were collected [55]. Worm somatic soluble antigens (SAg) and excretory-secretory (ES) antigens of *T. spiralis* from various developmental stages were prepared as previously described [6, 61].

### Bioinformatics analysis of TsEasp

The complete TsEasp gene coding sequence was retrieved from GenBank (XM_003373265.1). The physicochemical and structural properties of TsEasp were predicted using bioinformatic software and online servers [22]. Physicochemical characteristics were analyzed with the Expasy ProtParam tool. The signal peptide cleavage site was predicted using SignalP-5.0. Transmembrane helices of TsEasp were predicted with TMHMM-2.0. Sequence alignment between TsEasp and aspartyl proteases from other *Trichinella* species/genotypes was performed using BioEdit software. The GenBank accession numbers of these *Trichinella* aspartyl proteases are as follows: *Trichinella* T8 (KRZ91872.1), *T. nativa* (KRZ52016.1), *Trichinella* T9 (KRX53032.1), *T. murrelli* (KRX37180.1), *T. britovi* (KRY55371.1), *T. patagoniensis* (KRY14614.1), *T. nelsoni* (KRX24123.1), *T. pseudospiralis* (KRZ21443.1), *T. zimbabwensis* (KRZ06354.1), *T. papuae* (KRZ78894.1), and *Trichinella* T6 (KRX85117.1). Additionally, two other *T. spiralis* aspartic proteases (TsASP1 GenBank: QOQ72494.1; TsASP2 GenBank: XP_003380300.1) were also included in alignment analysis when performing sequence alignment of the *Trichinella* aspartic protease superfamily. Phylogenetic analysis was conducted using MEGA 11.0, and a phylogenetic tree was constructed by the neighbor-joining (NJ) method [12]. The tertiary structure and enzymatic active sites of TsEasp were predicted using SWISS-MODEL and PyMOL software [41].

### Preparation of rTsEasp and anti-rTsEasp serum

Total RNA was extracted from ML using TRIzol reagent (Sangon Biotech, Shanghai, PR China). TsEasp-specific primers containing ***EcoR* I** and ***Sal* I** restriction sites (bold) were designed as follows: forward 5′–CCG**GAATTC**ATGAATGACAGTGGTCGTGAACC–3′ and reverse 5′–GC**GTCGAC**CTAAAGCGACGGGCAAAATCCAAT–3′. The full-length sequence of the TsEasp gene was amplified by polymerase chain reaction (PCR). The amplified product was cloned into the pET-28a vector (Novagen, Darmstadt, Germany) harboring an N-terminal histidine tag and transformed into *Escherichia coli* BL21 (Novagen) [41]. The expression of rTsEasp was induced with 0.5 mM isopropyl β-D-1-thiogalactopyranoside (IPTG) at 16 °C [13]. The recombinant TsEasp protein (rTsEasp) was purified using His-tagged Ni-NTA-Sefinose resin (Sangon Biotech, Shanghai, PR China). Protein expression was analyzed by SDS-PAGE and Western blotting [1, 63].

In all, 20 BALB/c mice were subcutaneously immunized with 20 μg/mL rTsEasp emulsified in complete Freund’s adjuvant. At 2 weeks after the primary immunization, 3 booster immunizations were performed with 20 μg/mL rTsEasp mixed with incomplete Freund’s adjuvant. At 2 weeks after the 4th immunization, tail blood samples were collected from the mice, and anti-rTsEasp immune serum was isolated by centrifugation [43].

According to previous reports, the IgG antibody titer of the immune serum was determined by a conventional indirect ELISA method [15, 49]. Briefly, 20 μg/mL rTsEasp was coated onto microplates at 4 °C overnight. After washing with phosphate-buffered saline containing 0.05% Tween-20 (PBST, pH 7.4), the plates were blocked with 5% skimmed milk at 37 °C for 2 h. Following washes, serially diluted anti-rTsEasp immune serum was added and incubated at 37 °C for 2 h, followed by incubation with horseradish peroxidase (HRP)-conjugated goat anti-mouse IgG (1:10000, Sangon Biotech) for 1 h. The reaction was developed using O-phenylenediamine (OPD, Sigma, St. Louis, MO, USA) and terminated with 2 M H_2_SO_4_. The optical density (OD) at 492 nm was measured using a microplate reader (Molecular Devices, Vienna, Austria) [6].

### Western blot analysis

Western blot analysis was performed according to previous studies [59]. After the soluble proteins of *T. spiralis* at different developmental stages and rTsEasp were separated by SDS-PAGE, the proteins were transferred onto a membrane (Millipore, Burlington, MA, USA) at 0.25 A for 90 min using a wet transfer system (Bio-Rad, Hercules, CA, USA). The membrane was blocked with 5% skim milk in TBST (Tris-buffered saline containing 0.05% Tween-20) at room temperature for 2 h, then incubated with 1:100 dilutions of various sera (anti-rTsEasp serum, mouse anti-*T. spiralis* infection serum at 35 dpi, and pre-immune serum) at 4 °C overnight. After washing, the membrane was incubated with 1:10 000 HRP-conjugated goat anti-mouse IgG (Sangon Biotech, Shanghai, PR China) at 37 °C for 1 h. Signals were visualized using 3,3′-diaminobenzidine tetrahydrochloride (DAB, Solarbio, Beijing, PR China), and the reaction was terminated by rinsing with deionized water [26, 65].

### RT-PCR and qPCR

*Trichinella spiralis* worms at different developmental stages (ML, IIL, 3 d AW, 6 d AW, and NBL) were collected. Total RNA from various stages was isolated using TRIzol reagent (Sangon Biotech). RT-PCR was employed to determine the transcriptional level of TsEasp mRNA at different developmental stages of *T. spiralis* [57]. The housekeeping gene Glyceraldehyde 3 phosphate dehydrogenase (GAPDH) of *T. spiralis* (GenBank: AF452239) was amplified as an internal control.

Total RNA was extracted from diverse *T. spiralis* stages and reverse-transcribed into cDNA [34]. Specific primers for the *TsEasp* gene were designed using Primer Premier 5 (5′–GATACCGTCTGCATAGCTG–3′; 5′–CTGGTGGTGTAGAACGAGA–3′). The transcriptional levels of TsEasp at different developmental stages were quantitatively determined by qPCR using a 7,500 Fast Real-time PCR system (Thermo Fisher Scientific, Waltham, MA, USA) as described previously [40, 48].

### Indirect immunofluorescence test (IIFT)

To investigate the expression of native TsEasp in *T. spiralis* at different developmental stages, IIFT was performed [14, 27]. Briefly, the worms were fixed in cold acetone for 20 min and blocked with 1% BSA for 1 h. After washing with PBS, the worms were incubated with anti-rTsEasp serum, infection serum, and pre-immune serum (1:10) at 37 °C for 2 h, respectively. Then, the worms were then stained with goat anti-mouse IgG-Alexa Fluor 488 (1:100; Abways, Shanghai, China) at 37 °C for 1 h, and finally observed under a fluorescence microscope (Olympus, Tokyo, Japan).

To further explore the tissue localization of TsEasp in *T. spiralis*, worms at different stages (ML, IIL, and AW) were fixed with 4% paraformaldehyde, embedded in paraffin, and sectioned into 2-μm-thick slices using a microtome [12]. The sections were blocked with 5% goat serum at 37 °C for 1 h. After washing three times with PBS, the sections were incubated with anti-rTsEasp serum, infection serum, and pre-immune serum (1:10), respectively. Goat anti-mouse IgG-Alexa Fluor 488 (1:100; Abways) was used as the secondary antibody. After washing again, the worm sections were observed under a fluorescence microscope (Olympus) [67].

Moreover, binding and localization of rTsEasp and Caco-2 cells was also analyzed by IIFT [41]. First, Caco-2 cells were cultured in DMEM at 37 °C with 5% CO_2_ until they reached 90% confluence on coverslips. IIL soluble protein was used as the positive control, and PBS as the negative control. After washing with PBS, the cell monolayers were fixed with 4% formaldehyde for 20 min, followed by permeabilization with 0.25% Triton X-100 for 10 min. The cell monolayers were incubated with anti-rTsEasp serum, infection serum, and pre-immune serum at 4 °C overnight, and then incubated with anti-mouse IgG-Alexa Fluor 488 conjugate at 37 °C for 1 h. Finally, the cell nuclei were stained with 4′, 6-diamidino-2-phenylindole (DAPI) and observed under a microscope.

### Far-western blotting

To investigate the *in vitro* binding and interaction between rTsEasp and Caco-2 cells, soluble proteins extracted from Caco-2 and C2C12 cells were separated by SDS-PAGE [51]. The proteins were then electrotransferred onto a nitrocellulose (NC) membrane (Merck Millipore, Billerica, MA, USA). After blocking with skimmed milk in PBST at 37 °C for 1 h, the membrane was cut into strips, which were incubated with 20 μg/mL rTsEasp, ML soluble proteins, or BSA at 37 °C for 1 h [42]. After washing, the strips were probed with anti-rTsEasp serum, infection serum, or pre-immune serum diluted at 1:100 at 37 °C for 1 h. The strips were then incubated with horseradish peroxidase (HRP)-conjugated goat anti-mouse IgG for 1 h, followed by color development with DAB substrate (Solarbio). rTsEasp binding Caco-2 cell protein bands were analyzed using AlphaView software (AIC).

### *In vitro* larva invasion assay

To investigate the effects of rTsEasp protein and anti-rTsEasp serum on larval invasion of Caco-2 cell monolayers, ML were activated to IIL using 5% porcine bile from a pig slaughterhouse for subsequent invasion assays [27, 67]. Caco-2 monolayers were overlaid with 100 IILs suspended in semisolid medium supplemented with different concentrations of rTsEasp (0, 5, 10, 15, 20, and 25 μg/mL), or with anti-rTsEasp serum, infection serum, or pre-immune serum at serial dilutions from 1:100 to 1:1600. The cultures were incubated at 37 °C under 5% CO_2_ for 2 h. IIL ESP and BSA were used as positive or negative control. Furthermore, heat-inactivated rTsEasp and rTsEasp+Pepstatin A (aspartyl protease inhibitor) were used to determine the effect of rTsEasp enzymatic activity on larval invasion. Additionally, the number of larvae that invaded the cell monolayers was observed and counted under an inverted phase-contrast microscope (Olympus). Invaded larvae exhibited active movement within the monolayers, whereas non-invaded larvae remained suspended in the medium with a spiral coiling pattern. Each invasion assay was performed in triplicate.

### RNA interference (RNAi)

Three pairs of specific dsRNA primers were designed according to the cDNA sequence of the TsEasp gene, with sequences listed below: 5′–GATCAC**TAATACGACTCA CTATAGGG**TATTTGGCAGAAATTGGTGTGG–3′, 5′–GATCAC**TAATACGACTCACTA TAGGG**CTAAACCGCATATGCCATCTAA–3′; 5′–GATCAC**TAATACGACTCA CTATAG GG**TATGGTACTGGCAGTGTAGAAGG–3′, 5′–GATCAC**TAATACGACTCACTATAGGG** ACTGGACCGTGAATGAAAGATG–3′; 5′–GATCAC**TAATACGACTCACTATAGGG**GC TGGTTACTGGATGTTTGATATG–3′, 5′–GATCAC**TAATACGACTCACTATAGGG**CGA CGGGCAAAATCCAATTCTT–3′. Primers targeting green fluorescent protein (GFP) were designed and served as the negative control, and their sequences were as follows: 5′–GATCAC**TAATACGACTCACTATAGGG** TCCTGGTCGAGCTGGACGG–3′ and 5′–GATC AC**TAATACGACTCACTATAGGG** CGCTTCGTTGGGGTCTTTG–3′ [62]. The underlined letters are the protective bases, and the bold letters are the T7 promoter sequences. dsRNA primers were synthesized by Sangon Biotech. Various kinds of dsRNA were delivered into the ML via electroporation using an electroporator (Bio-Rad). Subsequently, transfected larvae were incubated in RPMI 1640 medium at 37 °C under 5% CO_2_ for 1–3 days. Larval survival rates in each group were assessed to identify the optimal dsRNA kind, concentration, and culture duration to verify silencing specificity. qPCR and Western blotting were performed to assess the levels of TsEasp mRNA transcriptional and protein expression, respectively, based on the previously described protocols [63]. GAPDH was selected as the internal reference gene for normalization. ImageJ software was utilized for grayscale analysis to quantify the relative expression of TsEasp protein.

### Statistical analysis

All data were analyzed using SPSS 27.0 software and expressed as the mean ± standard deviation (SD). One-way ANOVA and χ^2^ tests were performed to assess the statistical differences.

## Results

### Bioinformatic analysis of TsEasp

**Figure 1:**
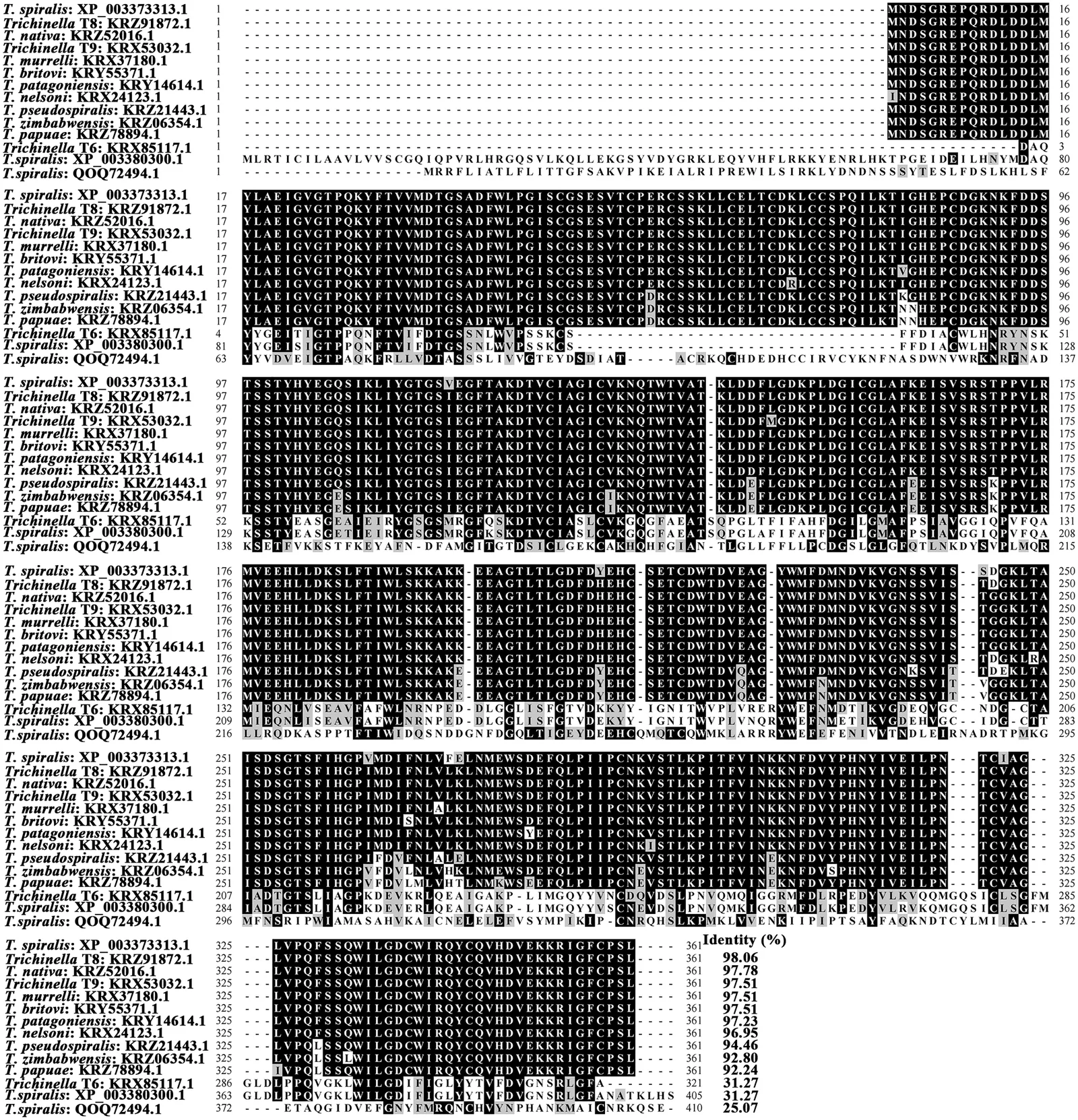
Sequence alignment of the *T. spiralis* eukaryotic aspartyl protease superfamily (XP_003373313.1) with other *Trichinella* species/genotypes. Black shading indicates residues identical to TsEasp, and conservative substitutions are shaded grey.

**Figure 2:**
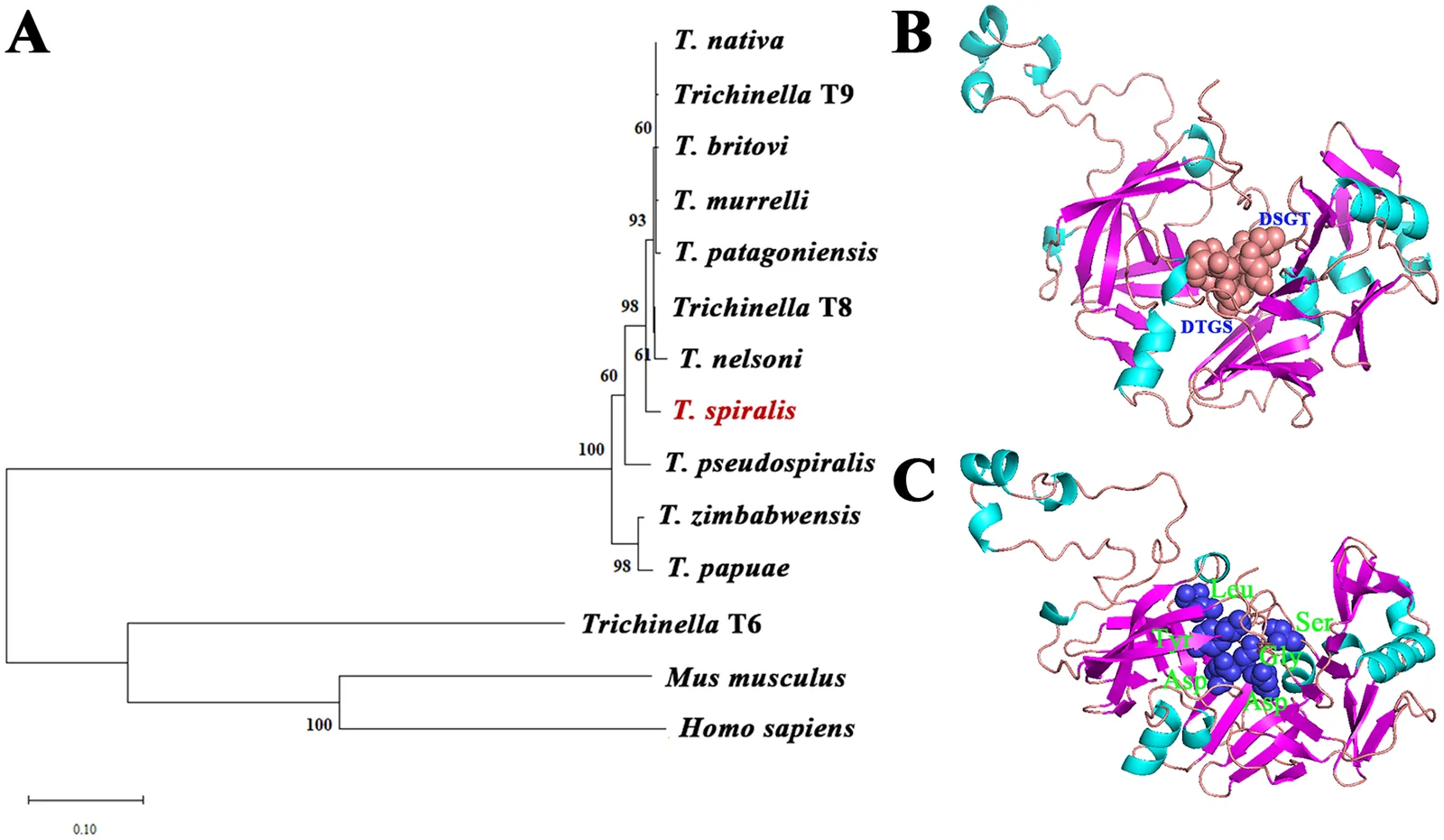
Phylogenetic tree analysis with the NJ method mapped with MEGA (A) and Predicted 3-dimensional structure (B, C) of the TsEasp protein. B: The pink spherical structure is the sequence of the active site of aspartic protease; N-terminal: DTGS, C-terminal: DSGT; C: The dark blue spherical structure is the binding site for the aspartic protease inhibitor, namely Asp35, Tyr112, Leu153, Asp253, Gly255, and Ser257. Blue represents the α-helix, purple the β-sheet, and pink the random coil.

Bioinformatics analysis showed that the full-length *TsEasp* gene was 1,086 bp, encoding a protein of 361 amino acids with a molecular weight of 40.25 kDa and a theoretical isoelectric point of 4.77. Online prediction using TMHMM-2.0 and SignalP-5.0 revealed that rTsEasp contained no signal peptide or transmembrane domain. Homology analysis between rTsEasp and aspartic proteases from other *Trichinella* species/genotypes is shown in Figure 1. The TsEasp amino acid sequence shared 98.06, 97.78, 97.51, 97.51, 97.51, 97.23, 96.95, and 31.27% identity with aspartic proteases from eight encapsulated *Trichinella* species/genotypes (*Trichinella* T8, *T. nativa*, *Trichinella* T9, *T. murrelli*, *T. nelsoni, T. britovi*, *T. patagoniensis,* and *Trichinella* T6), respectively, and 94.46, 92.80, and 92.24% identity with those from three non-encapsulated *Trichinella* species (*T. pseudospiralis, T. zimbabwensis*, and *T. papuae*), respectively. Moreover, the TsEasp amino acid sequence had 31.27 and 25.07% identity with those of TsASP1 and TsASP2, further confirming that TsEasp is a novel *T. spiralis* aspartyl protease that is different from previously reported TsASP1 and TsASP2. Phylogenetic analysis indicated that TsEasp was closely related to *Trichinella* T8, *T. nativa*, *Trichinella* T9*, T. murrelli, T. britovi*, *T. patagoniensis*, and *T. nelsoni* within the genus *Trichinella* (Fig. 2A). Functional prediction suggested that this protein is a pepsin-like aspartic protease with the active site motifs DTGS and DSGT, representing a bilobed enzyme capable of cleaving peptide bonds at acidic pH (Figs. 2B and Fig. 2C).

### Expression and antigenic identification of rTsEasp

**Figure 3:**
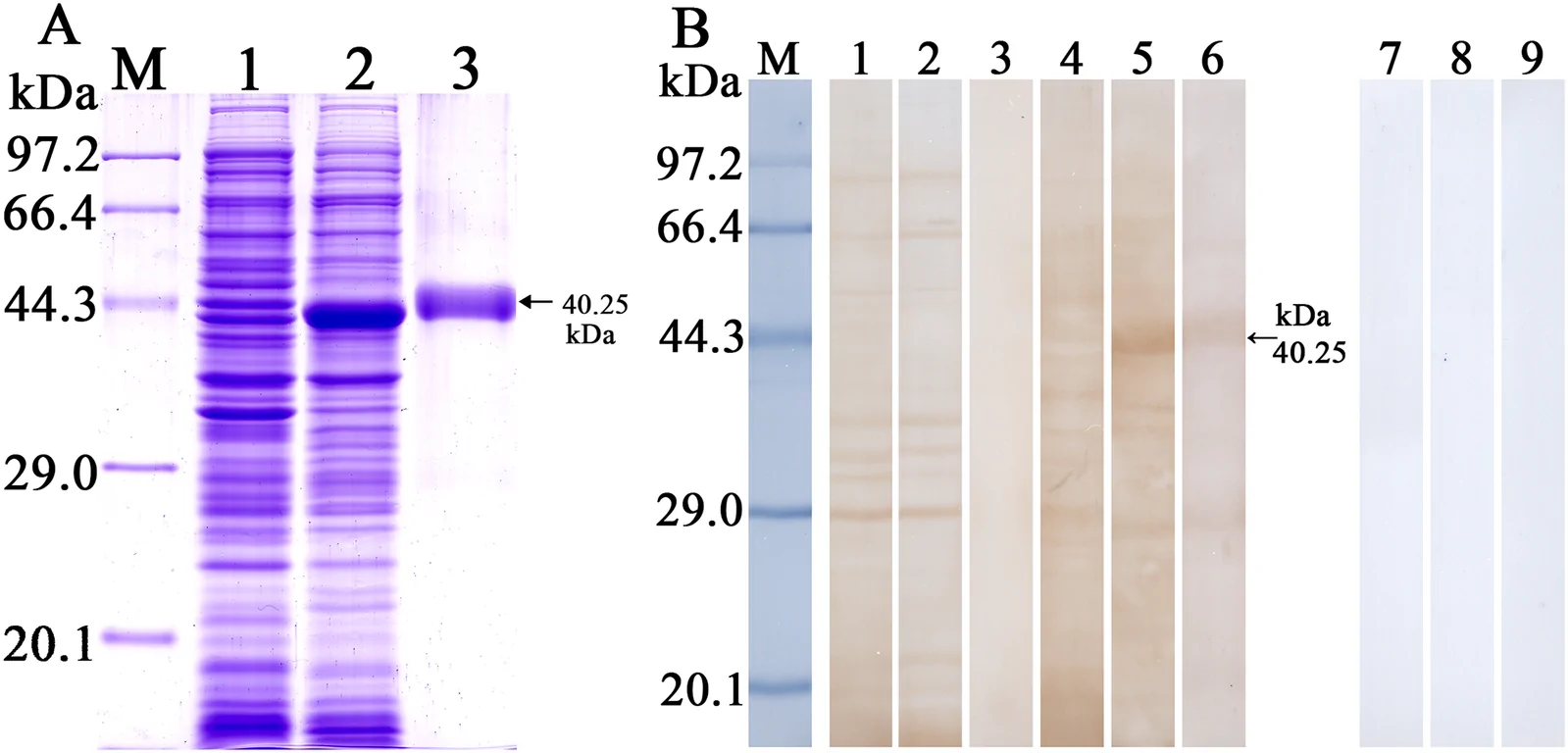
Expression and identification of rTsEasp. A: SDS-PAGE analysis of rTsEasp. Lane M: Protein marker; Lane 1: lysate of bacteria containing pET-28a/TsEasp before induction; Lane 2: lysate of induced bacteria containing pET-28a/TsEasp; Lane 3: purified rTsEasp. B: Western blotting of rTsEasp. The lysates of pET-28a/TsEasp prior to induction (lanes 1 and 7), the lysates of pET-28a/TsEasp (lanes 2 and 8) and purified rTsEasp (lanes 3 and 9) were not recognized by infection serum (lanes 1, 2, and 3) and pre-immune serum (lanes 7, 8, and 9). The lysates of pET-28a/TsEasp prior to induction (lane 4) were not recognized by anti-rTsEasp serum. The lysates of pET-28a/TsEasp (lane 5) and purified rTsEasp (lane 6) were probed by anti-rTsEasp serum.

After induction with IPTG, the histidine-tagged recombinant TsEasp protein was expressed and purified. SDS-PAGE analysis revealed a distinct band for rTsEasp, with a relative molecular mass of 40.25 kDa consistent with its predicted size (Fig. 3A). To evaluate antibody response elicited by rTsEasp immunization, anti- rTsEasp IgG titer was assayed by ELISA at 2 weeks after the 4th immunization. The specific IgG titer of immune serum was 1:10^5^, demonstrating that the rTsEasp had good antigenicity.

Western blot analysis showed that the lysates of pET-28a/TsEasp and purified rTsEasp were recognized by anti-rTsEasp serum; whereas the uninduced bacterial lysate containing pET-28a/TsEasp was not recognized, indicating that rTsEasp protein was successfully expressed in the prokaryotic expression system, and the expressed protein exhibited specific immunoreactivity, no specific binding was observed in the uninduced lysate control (Fig. 3B).

**Figure 4:**
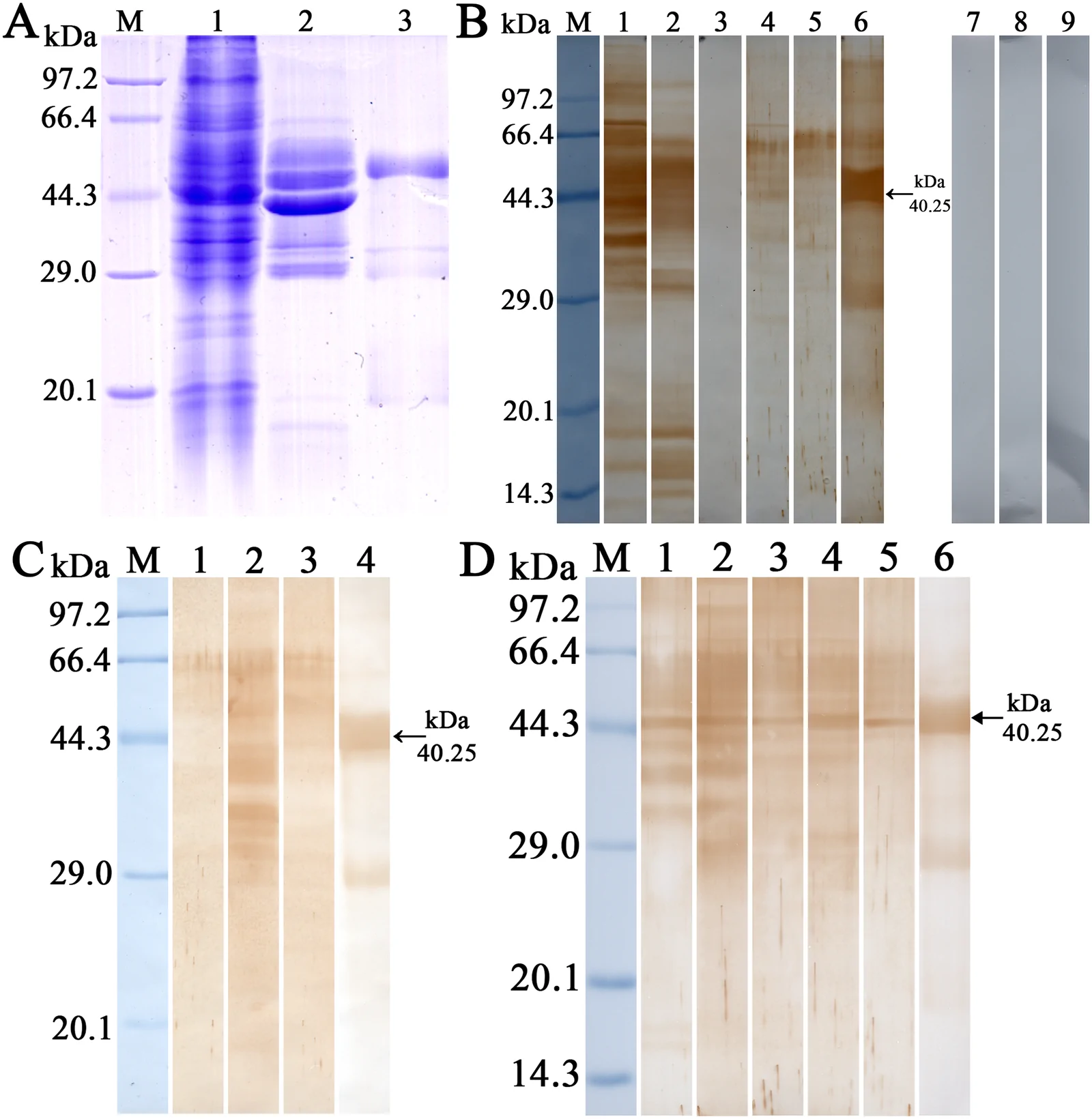
Antigenicity analysis of rTsEasp. A: SDS-PAGE analysis of ML crude and ES protein, and rTsEasp. Lane M: protein marker; Lane 1: ML soluble crude protein; Lane 2: ML ES protein; Lane 3: purified rTsEasp. B: Western blotting of rTsEasp. ML soluble protein (lanes 1, 4, and 7) were probed by *T. spiralis*-infected mouse serum (lane 1) and anti-rTsEasp serum (lane 4), but not pre-immune serum (lane 7); ML ES protein (lanes 2, 5, and 8) were recognized by infection mouse serum (lane 2), but not by anti-rTsEasp serum (lane 5), and pre-immune serum (lane 8). Purified rTsEasp (lanes 3, 6, and 9) were probed by anti-rTsEasp serum (lane 6), but not by infection mouse serum (lane 3) and pre-immune serum (lane 9). C: Western blotting showed that native TsEasp in ES proteins of ML (lane 1), IIL (lane 2), and AW (lane 3) were not detected by anti-rTsEasp serum, but rTsEasp were probed by anti-rTsEasp serum (lane 4). D: Western blotting revealed that native TsEasp in soluble proteins of ML (lane 1), IIL (lane 2), 3d AW (lane 3), 6d AW (lane 4), NBL (lane 5), and rTsEasp (lane 6) were recognized by anti-rTsEasp serum.

The ML soluble crude proteins, ML ES proteins, and rTsEasp were separated by SDS-PAGE and subjected to Western blot analysis. The results showed that anti-rTsEasp serum recognized native TsEasp with 40.25 kDa in ML soluble proteins, but not in ES proteins (Fig. 4). When anti-rTsEasp serum was used to detect soluble proteins from different *T. spiralis* stages, native TsEasp with 40.25 kDa was observed, but no native TsEasp with 40.25 kDa was detected in ES proteins from various stages, indicating that TsEasp is a surface and somatic soluble protein of the nematode, but not a secretory protein.

### TsEasp transcription at various *T. spiralis* stages

**Figure 5:**
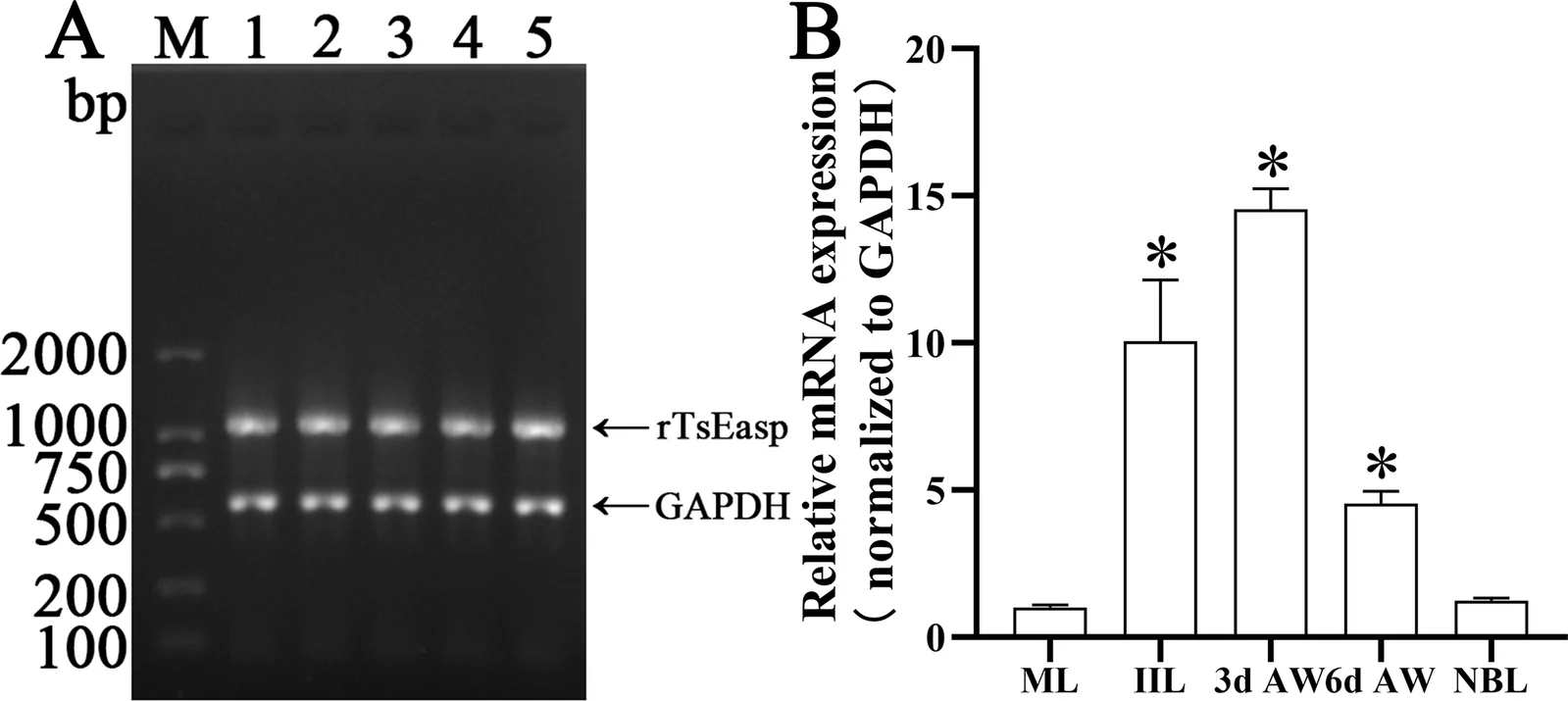
RT-PCR and qPCR analysis of TsEasp transcription in different *T. spiralis* phases. A: RT-PCR analysis. Lane M: DL 2000 DNA marker; Lane 1: ML; Lane 2: IIL; Lane 3: 3d AW; Lane 4: 6d AW; Lane 5: NBL. The arrows (←) indicate the positions of rTsEasp (1,086 bp) and GAPDH (570 bp). B: qPCR analysis. The transcription levels of TsEasp were calculated with the 2^−∆∆Ct^ method. GAPDH was utilized as an internal control. Asterisks (*) indicate significant differences compared with the ML stage (*p* < 0.05).

RT-PCR results showed that the *TsEasp* with a predicted size of 1,086 bp was detected in diverse lifecycle stage worms of *T. spiralis*, including ML, IIL, 3d AW, 6d AW, and NBL; the housekeeping gene (*GAPDH*) was also amplified in various stage worms as a positive control (570 bp) (Fig. 5A).

qPCR was used to quantitatively ascertain the transcriptional levels of the *TsEasp* gene at different *T. spiralis* developmental stages (ML, IIL, 3 d AW, 6 d AW, and NBL). The results showed that the *TsEasp* transcriptional levels at IIL, 3 d AW, and 6 d AW stages were significantly higher than those at the ML and NBL stages (*F* = 103.11, *p* < 0.01) (Fig. 5B).

### Expression and localization of TsEasp at diverse *T. spiralis* stages

**Figure 6:**
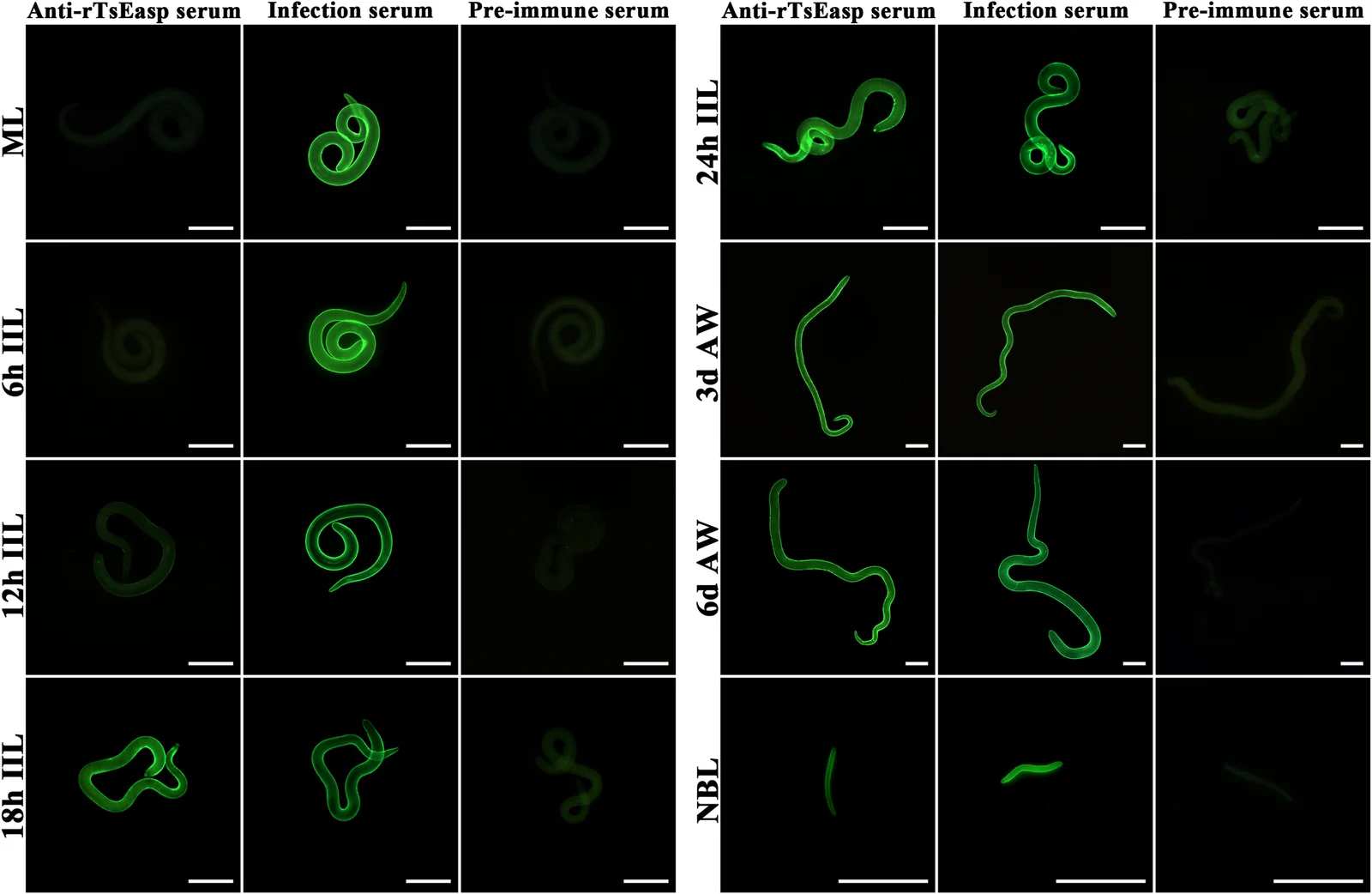
Expression of TsEasp at the surface of different *T. spiralis* stages by IIFT with anti-rTsEasp serum. Green fluorescence was detected in 18–24 h IIL, AW, and NBL stages, but not in ML and 6–12 h IIL stages. The worms were also probed by infection serum and pre-immune serum as a positive and negative control, respectively. Scale bars = 100 μm.

IIFT was performed to ascertain the TsEasp expression and localization in different developmental stages of *T. spiralis*. Immunofluorescence staining of whole worms using anti-rTsEasp serum showed that no fluorescent signal was detected on the surface of early-stage larvae (ML, 6 h and 12 h IIL), whereas positive immunofluorescence was observed in late-stage IIL (18 h and 24 h IIL) and adult worms (3 d and 6 d AW) (Fig. 6). These results indicated that TsEasp was not expressed in the cuticle of ML and early IIL, but was expressed in the cuticle of late IIL, AW, and NBL.

**Figure 7:**
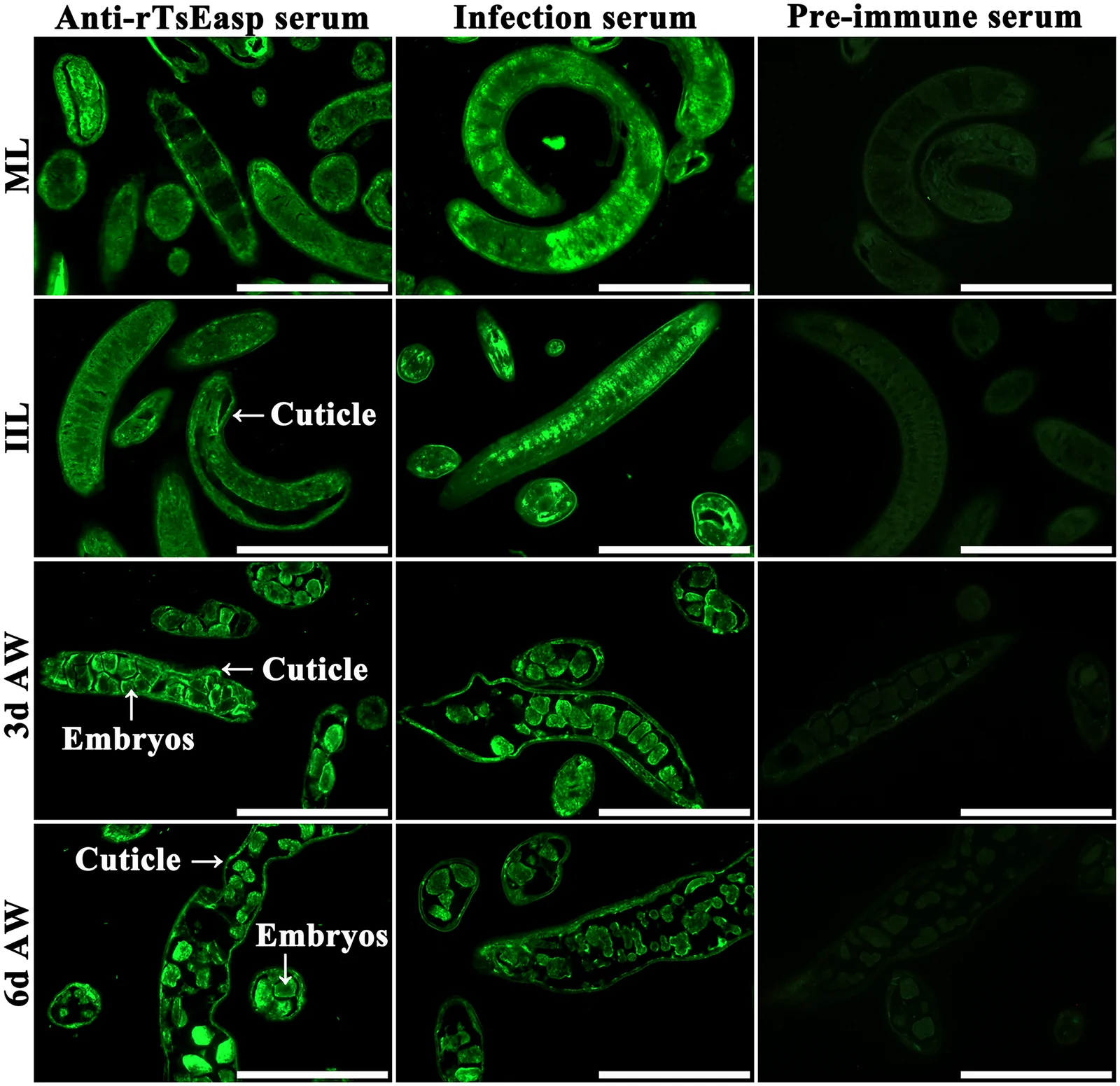
Expression and localization of TsEasp in various *T. spiralis* stages by IIFT with anti-rTsEasp serum. Immunofluorescence staining was mainly localized in the cuticle and around the embryos in female uterus. Worms incubated with infection serum and pre-immune serum served as the positive and negative control. Scale bars: 50 μm.

When the IIFT test was performed using worm tissue sections of different developmental stages, TsEasp was mainly localized in the cuticle, and around embryos in the uteri of female adult worms (Fig. 7).

### Far-western blotting analysis of rTsEasp binding to Caco-2 cells

**Figure 8:**
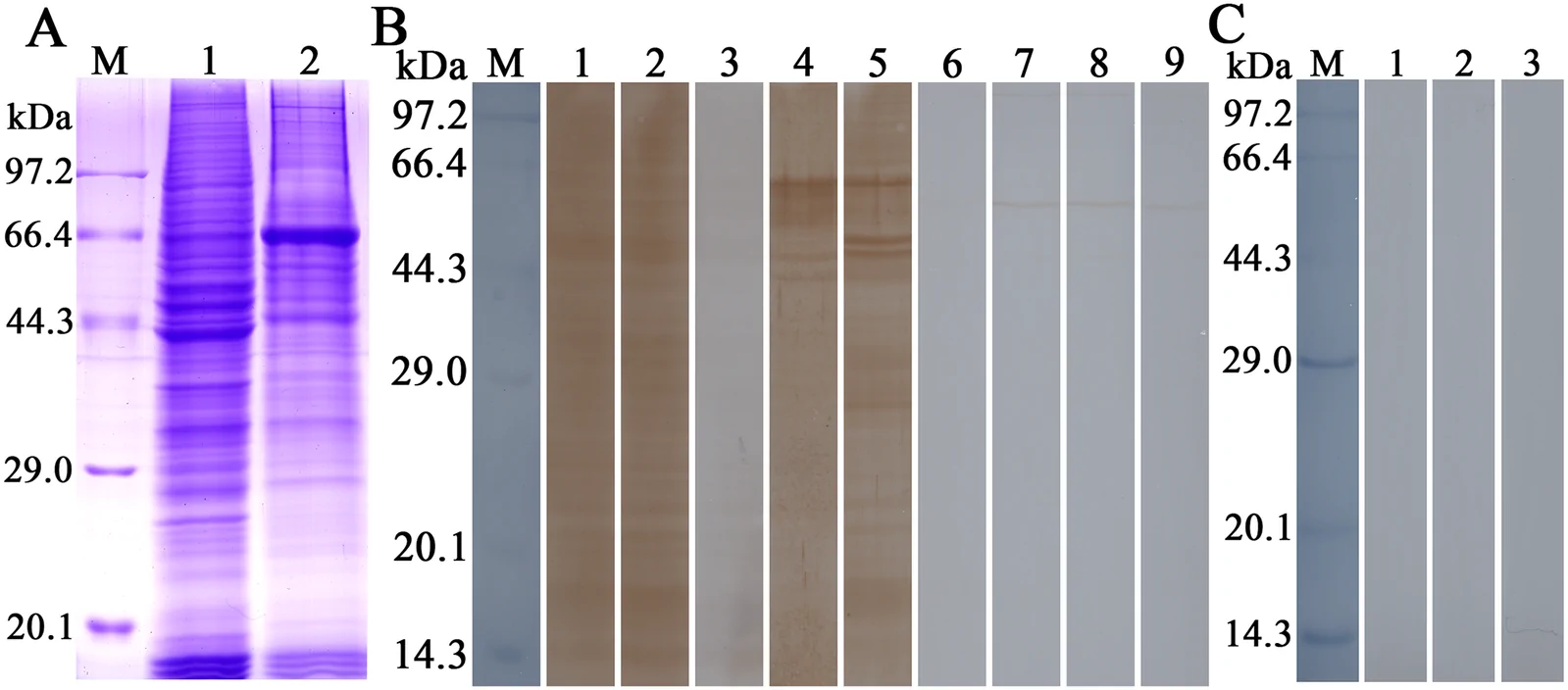
Far-western blotting of binding between rTsEasp and Caco-2. A: SDS-PAGE of Caco-2 (lane 1) and C2C12 (lane 2) cell lysates; Lane M: protein marker. B: Far-western blotting of Caco-2 proteins bound with rTsEasp. The Caco-2 proteins were firstly pre-incubated with rTsEasp (lanes 1–3), ML soluble protein (lanes 4–6) or BSA (lanes 7–9), and subsequently probed with anti-rTsEasp serum (lanes 1, 4, and 7), infection serum (lanes 2, 5 and 8) or pre-immune serum (lanes 3, 6, and 9). C: Far-western blotting of C2C12 proteins bound with rTsEasp. The C2C12 protein (lanes 1–3) was first pre-incubated with rTsEasp, and no protein bands were recognized by anti-rTsEasp serum (lane 1), infection serum (lane 2), or pre-immune serum (lane 3). There was no binding between rTsEasp and C2C12 protein.

SDS-PAGE analysis showed that Caco-2 cell proteins displayed approximately 32 distinct bands with molecular weight ranging from 21.2 to 93.4 kDa (Fig. 8A). Far-western blotting demonstrated that after incubation with rTsEasp, approximately 15 bands (18.0–68.5 kDa) of Caco-2 cell proteins were recognized by anti-rTsEasp serum, and about 17 bands (17.8–83.5 kDa) were recognized by infection serum; whereas no bands were detected by pre-immune serum. After incubation with ML soluble proteins, anti-rTsEasp serum recognized 5 bands (42.8–62.2 kDa) of Caco-2 cell proteins and infection serum recognized 11 bands (18.3–62.6 kDa); no visible bands were detected by pre-immune serum. When Caco-2 proteins were incubated with BSA, a single band with 58.7 kDa was reacted with anti-rTsEasp serum, infection serum and pre-immune serum, it was likely attributable to non-specific reaction (Fig. 8B). No binding bands were detected between C2C12 proteins and rTsEasp (Fig. 8C). Collectively, these results confirmed the occurrence of specific binding and interaction between rTsEasp and Caco-2 cells.

### IIFT analysis of binding of rTsEasp with Caco-2

**Figure 9:**
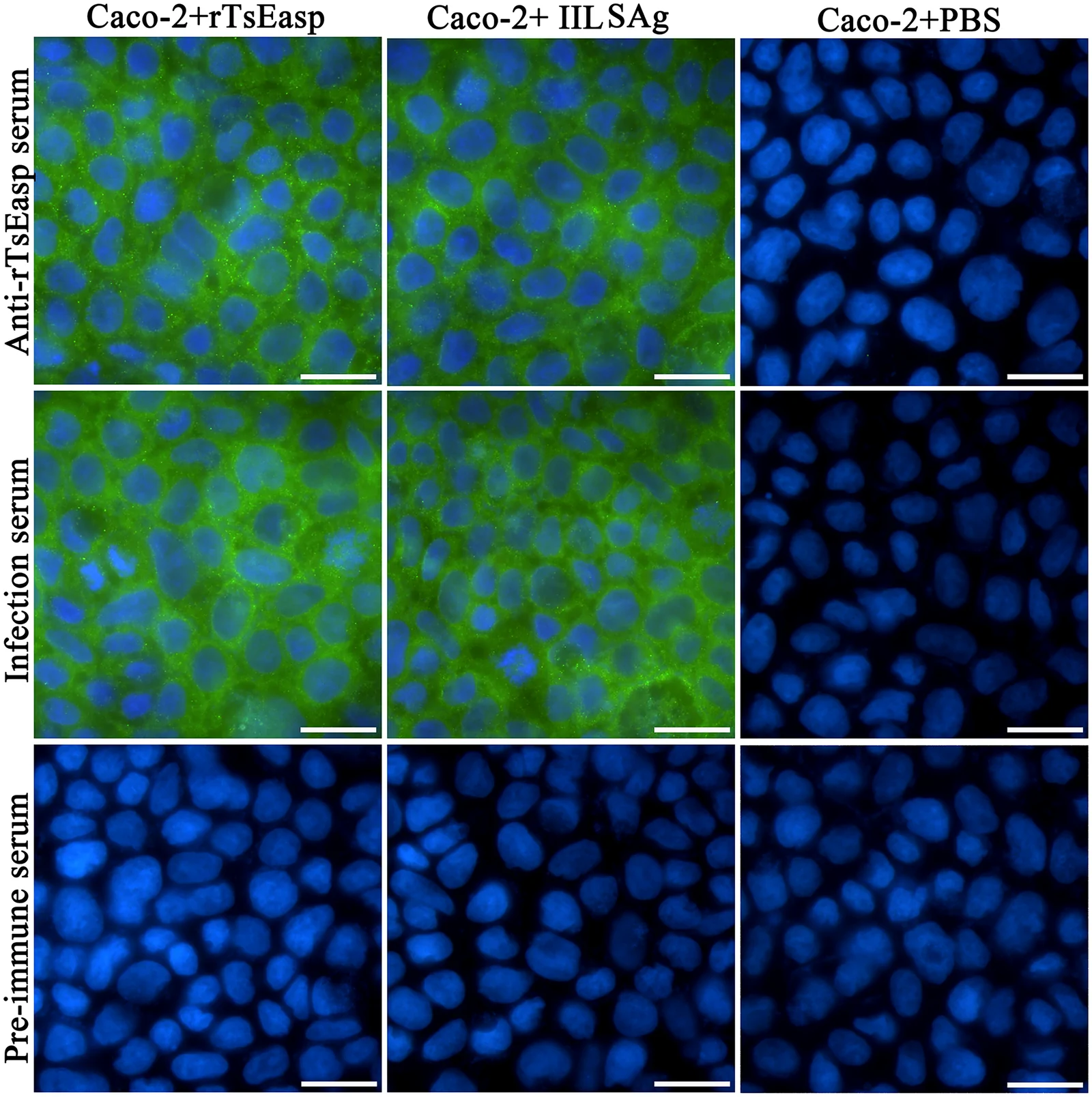
IIFT of binding of rTsEasp and Caco-2 cells. Caco-2 cells were first incubated with rTsEasp, IIL SAg, or PBS for 2 h at 37 °C. After incubation, the cells were probed by anti-rTsEasp serum, infection serum, or pre-immune serum, subsequently colored with goat anti-mouse IgG-488 conjugate. DAPI dyed cell nuclei in blue. Scale bars: 20 μm.

IIFI results showed that after Caco-2 cells were co-incubated with rTsEasp and IIL SAg, respectively, green fluorescence on the surface of Caco-2 cells was detected by both anti-rTsEasp serum and infection serum, but no fluorescent staining was observed in pre-immune serum group (Fig. 9). These results indicated that there is specific binding between rTsEasp and Caco-2 cells.

### rTsEasp promoting larval invasion and anti-rTsEasp antibody inhibiting larval invasion

**Figure 10:**
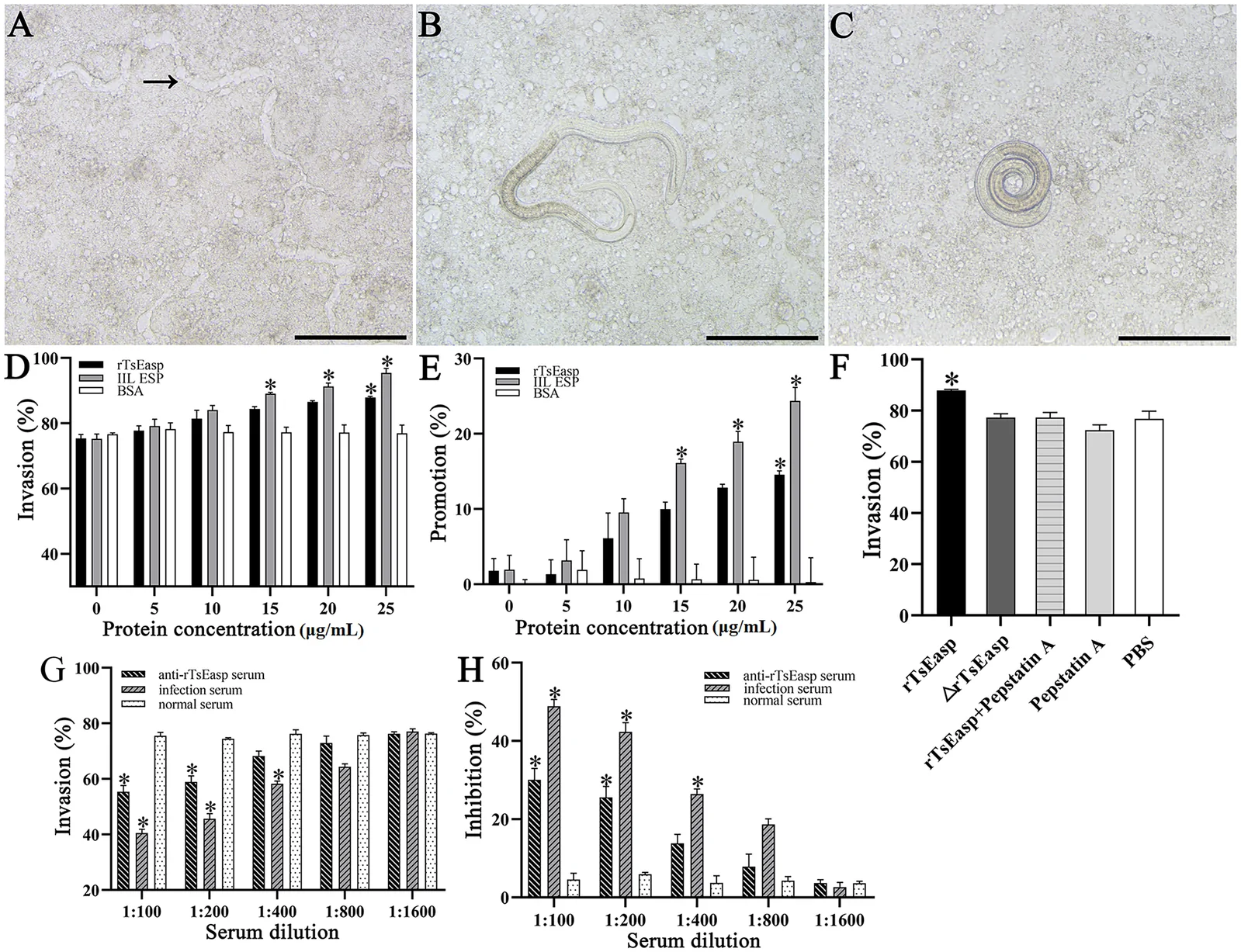
rTsEasp acceleration of larval invasion and anti-rTsEasp antibody suppression of invasion. After the IIL worms were added onto the Caco-2 monolayer, they were co-cultured at 37 °C with 5% CO_2_ for 2 h, and larval invasion was observed under a microscope. A: Larval migration tracks as shown by a black arrow. B: Larval invasion within Caco-2 cell monolayer. C: Non-invasive IILs suspended in the medium and coiled helically on the surface of cell monolayers. D and E: rTsEasp promotion of *T. spiralis* larval invasion of Caco-2 cell monolayers. F: Heat-inactivated rTsEasp and Pepstatin A-treated rTsEasp exhibited no promoting role on larval invasion. G and H: Different dilutions of anti-rTsEasp serum inhibited larval invasion. Scale bars: 200 μm. *p < 0.05 compared with BSA, PBS, or normal group.

Following the addition of IILs to Caco-2 cell monolayers, larvae invaded and migrated within the monolayers (Figs. 10A–10C). After the monolayers were treated with various concentrations of rTsEasp (0, 5, 10, 15, 20, and 25 μg/mL) for 2 h, the larval invasion rates were 75.33, 77.74, 81.43, 84.36, 86.55, and 87.91%, respectively. Larval invasion was gradually increased with higher rTsEasp concentration and in a dose-dependent manner (*F* = 30.148, *r* = 0.976, *p* = 0.001) (Fig. 10D). At a concentration of 25 μg/mL, the enhancement rate of larval invasion by rTsEasp was 14.57% compared with the PBS group (*χ*^2^ = 4.431, *p* = 0.023); in addition, BSA did not exhibit any promoting effect on larval invasion. (Fig. 10E). Meanwhile, incubation of IILs with heat-inactivated rTsEasp or rTsEasp treated with Pepstatin A showed no promoting effect on larval invasion of Caco-2 cells, compared to the rTsEasp group (Fig. 10F). Following treatment of the IILs with different dilutions of anti-rTsEasp serum for 2 h, the larval invasion was 55.31, 58.90, 68.23, 72.94, and 76.24%, respectively. Anti-rTsEasp serum inhibited larval invasion of Caco-2 monolayers, and the invasion was decreased with the increase of anti-rTsEasp serum dilution (*F* = 58.812, *r* = 0.985, *p* = 0.02). When immune serum dilutions at 1:100 and 1:200 were used, the inhibition rates of anti-rTsEasp serum were 30.07 and 25.58%, respectively (χ^2^_1:100_ = 9.192, *p* = 0.02; χ^2^_1:200_ = 5.747, *p* = 0.012). These findings indicate that rTsEasp facilitated larva penetration of Caco-2 monolayer, and anti-rTsEasp antibody impeded larval penetration.

### RNAi silencing the *TsEasp* gene reducing TsEasp expression and inhibiting larval invasion

**Figure 11:**
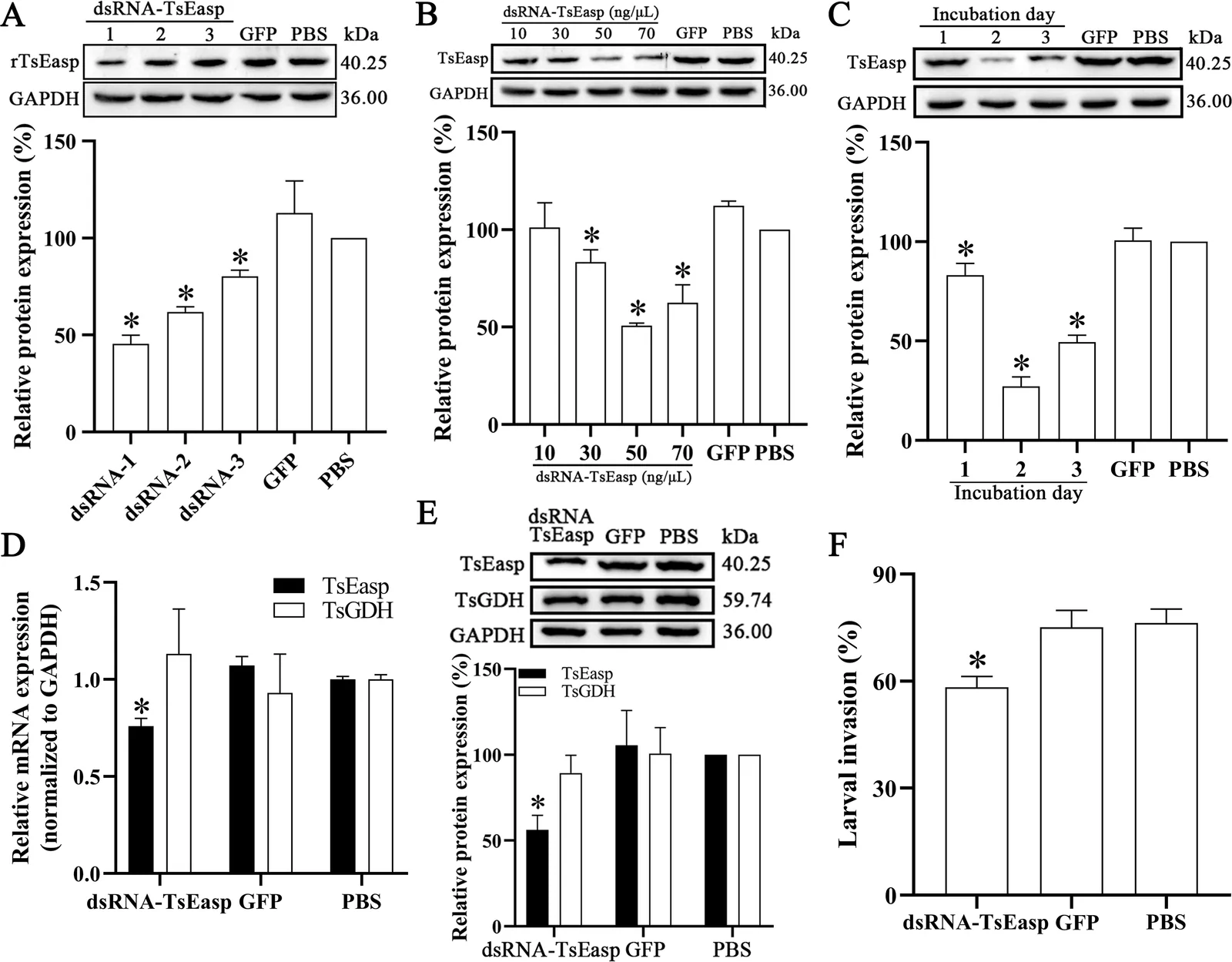
RNAi reduced TsEasp expression and larval invasion. A: Western blotting of TsEasp protein abundance after treatment with different kinds of dsRNA-TsEasp. B: Western blotting of TsEasp protein abundance following treatment with diverse concentrations of dsRNA-TsEasp-1. C: Western blotting of TsEasp protein abundance at various incubation times post dsRNA-TsEasp-1 delivery. D: qPCR analysis of TsEasp and TsGDH transcript levels for specificity verification. E: Western blotting of TsEasp and TsGDH protein levels for specificity verification. F: TsEasp-specific dsRNA suppressed larval invasion of Caco-2 monolayer. *p < 0.05 compared to the PBS group.

*Trichinella spiralis* ML were randomly allocated into five groups: dsRNA-TsEasp-1, dsRNA-TsEasp-2, dsRNA-TsEasp-3, dsGFP negative control, and PBS blank control group. Larvae were subjected to electroporation and subsequently cultured for 1–3 days. The larval survival rates of each group were 90.93, 92.41, 92.28, 90.09, and 91.09%, respectively (*F* = 0.6847, *p* = 0.6108), revealing that electroporation treatment exerted no significant influence on larval viability. Compared with the PBS group, TsEasp protein levels were reduced by 54.54, 38.13, and 19.75% in the dsRNA-TsEasp-1, dsRNA-TsEasp-2, and dsRNA-TsEasp-3 groups, respectively (*F* = 36.53, *p* < 0.0001) (Fig. 11A). No significant difference in TsEasp protein expression was observed between dsRNA-GFP negative control and the PBS group (*p* > 0.05). Collectively, these results confirmed that dsRNA-TsEasp-1 exhibited the strongest silencing efficiency. Therefore, dsRNA-TsEasp-1 was applied for all the subsequent experiments. Different concentrations (10, 30, 50, 70 ng/μL) of dsRNA-TsEasp-1 were delivered into the ML, and TsEasp protein abundance was determined following incubation for 2 days. Relative TsEasp protein levels in the 30, 50, and 70 ng/μL groups were decreased by 14.02, 45.46, and 40.14%, respectively (*F* = 36.05, *p* < 0.0001) (Fig. 11B). These data indicated that 50 ng/μL of dsRNA-TsEasp-1 represented the optimal dosage for RNAi treatment. Subsequently, 50 ng/μL of dsRNA-TsEasp-1 was transfected into the ML and incubated at 37 °C for 1, 2, and 3 days. Relative TsEasp protein abundance was reduced by 16.83, 72.87, and 50.59% after culture for 1, 2, and 3 days, respectively (*F* = 147.4, *p* < 0.0001) (Fig. 11C). Accordingly, 2 days was the optimal incubation duration for the *in vitro* RNAi. To verify the silencing specificity, the mRNA and protein levels of *T. spiralis* glutamate dehydrogenase (TsGDH, GenBank: XM_003380129.1) in the ML were observed after dsRNA-TsEasp-1 delivery. Neither transcriptional nor protein expression of TsGDH were significantly down-regulated (*p* > 0.05) (Figs. 11D and 11E). These findings confirm that dsRNA-TsEasp-1 specifically silences the TsEasp gene without obvious off-target effects. Finally, transfected larvae were overlaid onto Caco-2 monolayers and co-incubated for 2 h for evaluating larval invasion and migration. The larval invasion rates in the dsRNA-TsEasp group, GFP control group, and PBS control group were 58.30, 75.10, and 76.31%, respectively (Fig. 11F). Compared with the PBS group, the larval invasion inhibition rate of the dsRNA-TsEasp group was up to 23.55% (*F* = 25.96, *p* = 0.0002), indicating that silencing of the TsEasp gene significantly impairs the invasive capacity of *T. spiralis* larvae. These findings further demonstrate that TsEasp is an invasive protease in the process of *T. spiralis* infection.

## Discussion

Aspartic proteases are a class of endopeptidases activated under acidic conditions, which mediate proteolysis with water molecules as the nucleophilic center and are widely distributed in eukaryotes and microorganisms. Most of the aspartic proteases are synthesized as zymogens and exert their biological functions following activation, primarily involved in various physiological processes such as protein degradation, nutrient acquisition, antigen processing, zymogen activation, and signal transduction [3]. Members of the aspartic protease family exhibit high functional diversity; they are not only implicated in normal physiological activities, but also play crucial roles in pathogen invasion of hosts and virulence regulation.

Previous studies have demonstrated that *Toxoplasma gondii* aspartic proteases possess the typical catalytic properties of aspartic proteases and mediate host cell invasion and intracellular nutrient metabolism [66]. Aspartic proteases from *Plasmodium* spp. are involved in the cascade reaction of hemoglobin degradation and serve as key molecules of parasites to acquire nutrients [7]. Cathepsin D from *Schistosoma* spp. contributes to host hemoglobin degradation, nutrient uptake, growth, development and reproduction, and it also acts as an early diagnostic antigen and a target for therapeutic intervention [9, 18, 31]. The aspartic protease of *Necator americanus* is capable of degrading human hemoglobin and serum proteins under acidic conditions, and its specific antibody significantly inhibits larval skin penetration, rendering it an important candidate antigen for anti-hookworm vaccines [52]. Cathepsin E is a key molecule in host defense that regulates the expression of toll-like receptors (TLRs), enhances phagocytic bactericidal activity, participates in antigen presentation, induces tumor cell apoptosis, and maintains mucosal barrier integrity. It plays important roles in innate immunity, adaptive immunity, anti-tumor responses, and mucosal homeostasis [60].

*Trichinella spiralis* aspartic protease 1 (TsASP1) is localized in the stichosomes of the ML and IIL stages, as well as in myocytes and the periembryonic region within the female worm uterus. This TsASP1 is closely involved with *T. spiralis* development and survival in the host [59]. Another aspartic protease (TsASP2), which is present in *T. spiralis* ES products, exhibits specific hydrolytic activity on hemoglobin, collagen, and IgM under acidic conditions. Furthermore, TsASP2 acts as a key virulence factor to promote the *T. spiralis* invasion of host gut epithelial cells [56]. A recent study has demonstrated that TsASP2 directly hydrolyzed the tight junction proteins of gut epithelial cells, disrupted gut barrier integrity, and exacerbated colitis in mice [25]. However, no direct proteolytic activity of rTsEasp in this study was detected, despite the presence of a conserved aspartic protease active-site motif. The rTsEasp enzymatic activity was demonstrated indirectly through thermal inactivation and inhibition by inhibitor Pepstatin A. The absence of rTsEasp enzymatic activity can likely be attributed to inadequate post-translational modification, narrow substrate preference, or missing co-factors in the *in vitro* test [18].

In the present study, the full-length *TsEasp* coding sequence was cloned, and its recombinant protein was acquired by using a prokaryotic expression system. Bioinformatic analyses revealed that TsEasp is an acidic soluble protein devoid of signal peptides and transmembrane domains; it is highly conserved within the *Trichinella* genus and shares a close phylogenetic relationship with encapsulated *Trichinella* species. The TsEasp protein contains two canonical active motifs (DTGS and DSGT) and belongs to the pepsin-like acidic aspartic protease family [32]. RT-PCR and qPCR analyses demonstrated that TsEasp is transcriptionally expressed at all *T. spiralis* developmental stages, with significantly upregulated expression levels in IIL and AW stages. These findings suggested that TsEasp may be involved in *T. spiralis* development and parasitism in the host intestinal tract [24, 57]. IIFT results showed that TsEasp is expressed on the cuticle of late-stage IIL (18 h and 24 h IIL), adult worms, and NBL, but not in ML or early-stage IIL (6 h and 12 h IIL). Further localization observations on tissue sections of *T. spiralis* at different developmental stages confirmed that TsEasp is primarily distributed in the cuticle and periembryonic region of female adults. These data indicate that TsEasp mainly exerts its functions during the parasitic stage following *T. spiralis* invasion of the host intestine, and it may be involved in the nutrient acquisition and embryonic development of the parasite. SDS-PAGE and Western blot analyses showed that rTsEasp was successfully expressed and purified in the prokaryotic system. The protein had a molecular weight of 40.25 kDa, consistent with its predicted molecular weight. Furthermore, only the rTsEasp protein was specifically recognized by anti-rTsEasp serum, confirming that rTsEasp possesses favorable immunological specificity [21]. In addition, native TsEasp was detected in soluble proteins of *T. spiralis* at all developmental stages (ML, IIL, 3 d AW, 6 d AW, and NBL), but it was not detected in ES proteins. This result confirms that TsEasp is a somatic soluble protein of *T. spiralis* and is stably expressed throughout its entire developmental cycle [15, 43].

Far-western blotting has been widely used to assess the interactions between proteins [38]. In this study, we investigated the interaction between rTsEasp and Caco-2 cells, and the results showed that approximately 15 Caco-2 cell protein bands bound to rTsEasp. Herein, IIFT results demonstrated that rTsEasp could specifically bind to the surface of Caco-2 cells, suggesting that TsEasp is likely to participate in the *T. spiralis* invasion process by directly recognizing and binding to host intestinal epithelial cells.

Accumulating evidence has indicated that parasite surface proteins, which are directly exposed to the host immune system, serve as crucial target antigens. Specifically, some helminth surface proteins facilitate larval invasion, development, and survival through two main pathways: either by modulating host immune responses to achieve immune evasion, or by regulating host cell gene expression to reshape the microenvironment surrounding the parasite [23, 54, 58]. The *in vitro* invasion assays demonstrated that rTsEasp significantly enhanced the invasive capacity of *T. spiralis* larvae toward Caco-2 cell monolayers in a dose-dependent manner. Following heat denaturation or pepstatin A treatment, the facilitative role of rTsEasp on larval invasion was completely abrogated, indicating that the TsEasp role on larval invasion relies on its aspartic protease activity. Notably, no significant proteolytic activity of rTsEasp was detected in the present study, implying that its enzymatic activity may only be triggered under the host cell microenvironment or specific intracellular conditions within the parasite. Furthermore, anti-rTsEasp antibodies markedly impeded larval invasion, with the inhibitory effect intensified in an antibody dose-dependent manner. TsEasp-specific dsRNA also suppressed the larval invasion of the Caco-2 monolayer. Collectively, these findings demonstrate that TsEasp serves as a critical invasive virulence factor in *T. spiralis* invasion of the host intestinal epithelium and represents a promising target for intervention against *Trichinella* infection [28, 62].

However, this research has some limitations. Far-western blotting showed that TsEasp binds to multiple protein bands in Caco-2 cells, but the specific binding partners were not identified. The host’s target candidates should be identified and validated further by using a pull-down experiment coupled with mass spectrometry and co-immunoprecipitation test in a future study. The role of TsEasp in promoting *T. spiralis* invasion has thus far only been demonstrated at the *in vitro* cellular level. Its function has not yet been investigated *in vivo*, nor have studies been carried out on its potential as a target for intervention. As a result, it is difficult to comprehensively characterize its actual biological function in the host. Furthermore, the precise molecular mechanisms underlying TsEasp-mediated regulation of host immunity and intestinal epithelial damage remain to be elucidated. Future investigations employing experimental animal models and molecular interaction analyses are therefore warranted to further clarify the relevant mechanisms.

In conclusion, TsEasp is expressed throughout all *T. spiralis* developmental stages, with significantly upregulated expression levels in intestinal infective larvae and adult worms. TsEasp is predominantly distributed in the cuticle and periembryonic regions within the female uterus. rTsEasp specifically binds to the surface of Caco-2 cells and significantly enhances *T. spiralis* larval invasiveness in a dose-dependent manner, while anti-rTsEasp antibodies and RNAi effectively impair larval invasion. Accordingly, TsEasp is an important invasive virulence factor in *T. spiralis* infection.
